# Cerebellar Motor Learning: When Is Cortical Plasticity Not Enough?

**DOI:** 10.1371/journal.pcbi.0030197

**Published:** 2007-10-26

**Authors:** John Porrill, Paul Dean

**Affiliations:** Department of Psychology, Sheffield University, Sheffield, United Kingdom; University College London, United Kingdom

## Abstract

Classical Marr-Albus theories of cerebellar learning employ only cortical sites of plasticity. However, tests of these theories using adaptive calibration of the vestibulo–ocular reflex (VOR) have indicated plasticity in both cerebellar cortex *and* the brainstem. To resolve this long-standing conflict, we attempted to identify the computational role of the brainstem site, by using an adaptive filter version of the cerebellar microcircuit to model VOR calibration for changes in the oculomotor plant. With only cortical plasticity, introducing a realistic delay in the retinal-slip error signal of 100 ms prevented learning at frequencies higher than 2.5 Hz, although the VOR itself is accurate up to at least 25 Hz. However, the introduction of an additional brainstem site of plasticity, driven by the correlation between cerebellar and vestibular inputs, overcame the 2.5 Hz limitation and allowed learning of accurate high-frequency gains. This “cortex-first” learning mechanism is consistent with a wide variety of evidence concerning the role of the flocculus in VOR calibration, and complements rather than replaces the previously proposed “brainstem-first” mechanism that operates when ocular tracking mechanisms are effective. These results (i) describe a process whereby information originally learnt in one area of the brain (cerebellar cortex) can be transferred and expressed in another (brainstem), and (ii) indicate for the first time why a brainstem site of plasticity is actually *required* by Marr-Albus type models when high-frequency gains must be learned in the presence of error delay.

## Author Summary

Our ability to learn skilled movements depends crucially on the cerebellum, hence understanding cerebellar plasticity is central to theories of motor learning. The adaptation of the vestibulo-ocular reflex (VOR) is often used to test these theories. This reflex stabilizes the retinal image by moving the eyes to compensate for head movements signaled by the vestibular system. There is a long-standing puzzle in that classical Marr-Albus theories of VOR adaptation only require sites of plasticity in cerebellar cortex whereas experiment reveals plasticity in both cerebellar cortex and brainstem. We resolve this puzzle by showing that unavoidable delays in processing retinal slip severely limit cerebellar performance at high frequencies but that introducing a second brainstem site of plasticity driven by the correlation between cerebellar and vestibular inputs overcomes this limitation. Hence a second site of plasticity is *required* by Marr-Albus models for high-frequency learning in the presence of delay. The plasticity mechanism we describe is of general interest because it provides a biologically plausible route by which motor information learnt in one brain area (cerebellar cortex) can be transferred to and expressed in another area (the brain stem).

## Introduction

The cytoarchitecture of cerebellar cortex is remarkably uniform, suggesting that there is a single cerebellar algorithm which is applied to many different tasks, with each task specified by the connections of an individual cortical region [1–3]. This arrangement indicates the importance of modelling the algorithm, and also of choosing an appropriate task for subsequent model testing.

One of the most popular tasks, first proposed by Ito [1], has been calibration of the angular vestibulo-ocular reflex (VOR). The function of the VOR is to maintain a stable retinal image by counter-rotating the eyes in response to sensed movements of the head. Since the output of the reflex (eye movement) has no effect on its input (vestibular signals), it operates in feedforward or open-loop mode. Long-term calibration is therefore required to ensure accuracy, and extensive evidence implicates the floccular region of the cerebellum (i.e., flocculus and ventral paraflocculus) in this process (for a recent review, see Boyden, Katoh, and Raymond [4]).

**Figure 1:**
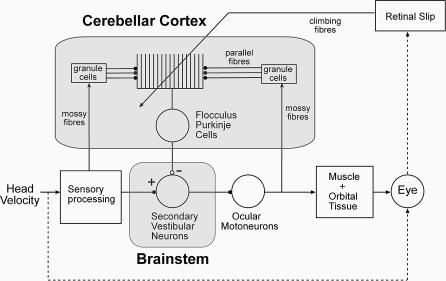
Simplified Diagram of the Circuitry That Mediates the Horizontal VOR Head-velocity signals are processed by the semicircular canals and primary vestibular neurons, relayed to secondary vestibular neurons in the brainstem, and then passed to ocular motoneurons (the classic 3-neuron arc). Motor command signals from the motoneurons control the oculomotor plant, i.e., eye muscles plus orbital tissue, in order to produce eye movements that counteract the effects of the head velocity on the retinal image. Inaccurate eye movements produce retinal slip, which is detected by the visual system. A side loop to the main 3-neuron arc passes through the floccular region of the cerebellum. This region of cerebellar cortex receives as mossy fiber input vestibular information and a copy of the motor command sent to the eye muscles. These mossy fiber inputs are converted into parallel fiber signals by granule cells and associated circuitry in the granular layer, and the parallel fiber signals influence simple spike firing (∼100 spikes/s) in Purkinje cells. Variation in simple spike firing is transmitted to a subset of secondary vestibular neurons (floccular target neurons) in the brainstem. The flocculus also receives a retinal-slip signal as climbing fiber input, which produces low-frequency (∼1 spike/s) complex spikes. Evidence from studies of VOR adaptation suggest that there are two sites of neural plasticity, one in cerebellar cortex and one in brainstem [8,44]. The simplified diagram omits cerebellar interneurons, and shows the efference copy of the motor commands as originating from the oculomotor neurons themselves. In reality this signal appears to originate from a number of areas, in particular the cell groups of the paramedian tracts [34–36].

Initial studies of horizontal VOR adaptation in primates suggested that the classical Marr-Albus theories of cerebellar learning [5,6] were incorrect [7]. These theories required a site of synaptic plasticity between parallel fibers and Purkinje cells, controlled by climbing fiber input that functioned as an error or teaching signal. However, the initial evidence appeared to point to a site of synaptic plasticity not in cerebellar cortex but in the brainstem (Figure 1). The subsequent controversy generated extensive experimental work, with a slowly emerging consensus that plasticity at *both* brainstem *and* cerebellar cortical sites is required for VOR adaptation [4,8,9]. Although this view does not directly falsify the classical theories, it does raise the question of *why* the cortical learning mechanisms they proposed should require supplementation by an extracerebellar site of plasticity. The adaptive-filter implementation of Marr-Albus theories (e.g., [10–12]) is a powerful signal processing device with no apparent need for an external site of plasticity, especially for what appears to be the straightforward task of learning a simple gain. The presence of brainstem plasticity suggests that a significant aspect of cerebellar function is not well-understood. It is therefore important to characterize the computational role of brainstem plasticity, in order both to clarify the capacities of cerebellar cortical circuitry, and to determine whether a brainstem site of plasticity is likely to be a functional requirement of VOR calibration alone, or a more general feature of cerebellar motor learning.

From the perspective of control theory, there are two features of image stabilization that together suggest a possible reason why a brainstem site of plasticity might be essential for VOR calibration. One is that processing of whole-image movement (usually referred to as retinal slip) takes ∼100 ms [13]. The implications of this delay for feedback control of image movement (the optokinetic reflex (OKR)) are well-known. Time delay in a negative feedback control loop causes stability problems whose solution necessarily leads to degraded tracking performance (p. 457 in [14]). In the case of the OKR, experimental data indicate that phase lag becomes severe above 1 Hz, and closed loop gain decreases to ∼0.5 at 2 Hz [15,16]. What is not so widely appreciated is that similar considerations would also apply if retinal slip was used as a teaching or error signal to calibrate a feedforward reflex such as the VOR, since the learning rules required in these models are also subject to instability when the teaching signal is delayed. Marr-Albus type models do indeed use retinal slip in this fashion, consistent with extensive evidence that the climbing fiber input to the floccular region of the cerebellum carries retinal slip signals [17,18]. The ∼100 ms delay in these climbing fiber signals [19,20] would appear to compromise learning at frequencies well below the ∼25 Hz at which the VOR reliably operates [21,22].

The second feature of the VOR relevant to a brainstem site of plasticity is the dynamics of the oculomotor plant (Figure 1). The oculomotor plant consists of the globe, its supporting tissues, and the extraocular muscles. It is primarily a viscoelastic system, with inertia playing a very minor role [23]. Control of such systems has two main aspects, one for high and one for low frequencies. At sufficiently high frequencies, a viscoelastic system responds to an input signal as a simple viscosity, so that its velocity is related to the input by a frequency-independent gain term with no phase shift. This is in contrast to its behavior at lower frequencies, where an increasing proportion of the input command is taken up by the plant's elasticity, resulting in reduced velocity gain and shifts in phase that are frequency-dependent (e.g., [24]). Calibrating the VOR in the face of plant changes therefore requires two components: (i) adjusting a simple gain to deal with plant viscosity at high frequencies, and (ii) altering a complex dynamic filter to deal with the effects of elasticity at lower frequencies.

The relevance of these two features for sites of plasticity in VOR calibration is that, although the complex dynamic filter would appear to be implemented by the microcircuitry of cerebellar cortex (see below), in principle a simple gain could be stored in the brainstem. It might therefore prove possible to meet the VOR's requirement for high-frequency performance combined with a delayed error signal for calibrating it, by allowing the cortex to learn the gain value first at intermediate frequencies, and then transfer it to the brainstem where it could be used at both intermediate and high frequencies. Such a mechanism would in principle be consistent with experimental observations that the VOR can only adapt to frequencies below ∼10 Hz [25], yet after adaptation can perform up to ∼25 Hz [21,22].

**Figure 2:**
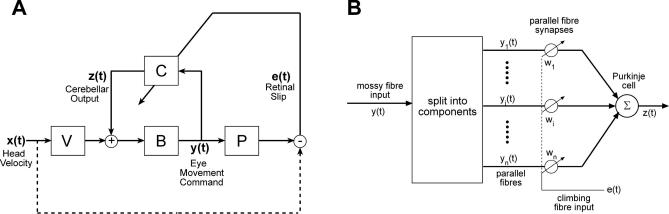
Linearised Model of Horizontal Vestibulo-Ocular Reflex, Derived from the Neural Circuitry Illustrated in Figure 1 (A) Head velocity *x*(*t*) is processed by the filter *V*, then added to the output *z*(*t*) of the adaptive filter *C* (which corresponds to the floccular region of cerebellum). The summed signal is then passed to the brainstem controller *B*. The output of *B* is a motor command *y*(*t*), which acts on the plant *P*. A copy of *y*(*t*) is sent back to the adaptive filter *C*. The command *y*(*t*) acts on *P* to move the eyes, a movement which is added to the head velocity *x*(*t*): net image movement is detected as retinal slip *e*(*t*) and sent to *C*. (B) Structure of the adaptive filter shown as *C* in (A). The copy of the eye-movement command *y*(*t*) arrives as mossy fiber input, and is decomposed into components *y_1_*(*t*) *.... y_n_*(*t*) by the granule cell layer. Each output component *y_i_*(*t*) is weighted by *w_i_*, corresponding to the efficacy of the corresponding synapse between a parallel fiber and the Purkinje cell. The weighted components are summed by the Purkinje cell and constitute the filter output. The value of each weight *w_i_* is adjusted according to the current value of the correlation between its component *y_i_*(*t*) and the global retinal slip signal *e*(*t*), which arrives as climbing fiber input.

We investigate this possibility by modelling the role of the cerebellum in VOR calibration, using a systems-level approach intended to expose the underlying structure of the problem by reducing it to its key signal-processing features. This approach has been used extensively in the oculomotor system to identify functional requirements that must operate whatever the details of the underlying neural circuits [26,27]. As applied here, its main features are:

(i) The dynamic components of the basic VOR circuitry shown in Figure 1 are linearised (Figure 2A). This approximation, likely to be reasonable for small-amplitude eye movements around the primary position, allows the use of powerful modelling and analytic techniques (cf. [28]). (ii) Cerebellar cortex is modelled as an adaptive linear filter (Figure 2B), the simplest version of Marr-Albus models suitable for dynamic processing [10,29], using a conventional covariance learning rule [30]. (iii) Only VOR calibration for changes in the oculomotor plant is simulated. Eye-movement inaccuracies in the VOR can arise from at least two sources: namely, changes in vestibular processing or changes in the mechanical properties of the oculomotor plant (i.e., extraocular muscles (EOMs) plus orbital tissues). In the former case, only some types of eye movement become inaccurate, so that the nature of the calibration required depends on factors such as the statistical proportions of different types of eye movement in a particular experimental situation, and the precise nature of the interaction in a given species between different eye-movement subsystems such as VOR and smooth pursuit or the OKR (see Discussion). Plant changes, in contrast, apply to all types of eye movement, so the required adaptive response is easier to analyze.

Previous work with this approach has shown how the adaptive filter version of the Marr-Albus model could be used to compensate for the complex low-frequency plant dynamics referred to above [11,12] . Here it is extended to examine the basis for multiple sites of plasticity, by simulating the effects of a realistic retinal-slip delay, and showing how they can be overcome for plausible oculomotor plants by storing a value related to high-frequency plant gain at a site outside cerebellar cortex. On this analysis, a brainstem site of plasticity is actually *required* by Marr-Albus theories, when operating under the particular conditions of VOR adaptation.

Part of this work has been reported previously in abstract form [31].

## Results

In the following simulations of plant compensation in the VOR, the cerebellar model (*C* in Figure 2A) is assumed to have only a single site of plasticity, corresponding to the synapses between parallel fibers and Purkinje cells (Figure 2B). The likely presence of additional plastic sites in cerebellar cortex does not affect the division of labor between cortex and brainstem that is demonstrated below.

The results are structured to show first that an adaptive filter is a powerful device that can cope with changes in both plant dynamics and high frequency gain, provided the error signal is not delayed. Next they show that delay in the error signal causes instabilities in learning above a certain frequency. These can be prevented by removal of high frequencies from the filter inputs, but learning at those frequencies is also prevented. Finally, they show that, although some improvements can be achieved with the eligibility trace, suitable high-frequency performance requires an algorithm for transferring a single gain value to a (brainstem) site outside the filter.

### Good Learning if Retinal Slip Is Not Delayed

Our previous investigations of VOR calibration [11,12] focused on low-frequency dynamics rather than on the high-frequency gain (Introduction). A fundamental difficulty faced by adaptive controllers in compensating for plant dynamics is that values for the correct motor commands are not known. The difference between correct and actual motor commands, the motor error signal, is therefore also unknown. Moreover, as noted in the Introduction, the climbing fiber input to the flocculus, which in Marr-Albus models constitutes the error signal, conveys sensory information about retinal slip rather than motor information about eye-movement commands [17,18]. It proved possible to show [11,12] that this difficulty could be overcome by giving the filter access to a copy of the commands sent to the EOMs as parallel fiber input (Figure 2A). A conventional cerebellar learning rule [30] could then be used to decorrelate motor commands from retinal slip, a procedure which mathematical analysis shows will converge to the correct solution for a very wide range of mechanical plants. The recurrent architecture needed to convey a copy of the motor commands as mossy fiber input to the floccular region (Figure 2A) is consistent with known anatomy and physiology [32–36], and the appropriate learning rule would appear to be that implemented by the recently described bidirectional plasticity at the parallel fiber—Purkinje cell synapse [37,38].

Here we emphasize that, provided the retinal slip signal is not delayed, a single site of plasticity in cerebellar cortex allows the algorithm to learn accurate plant compensation for both low-frequency dynamics and high-frequency gain.

**Figure 3:**
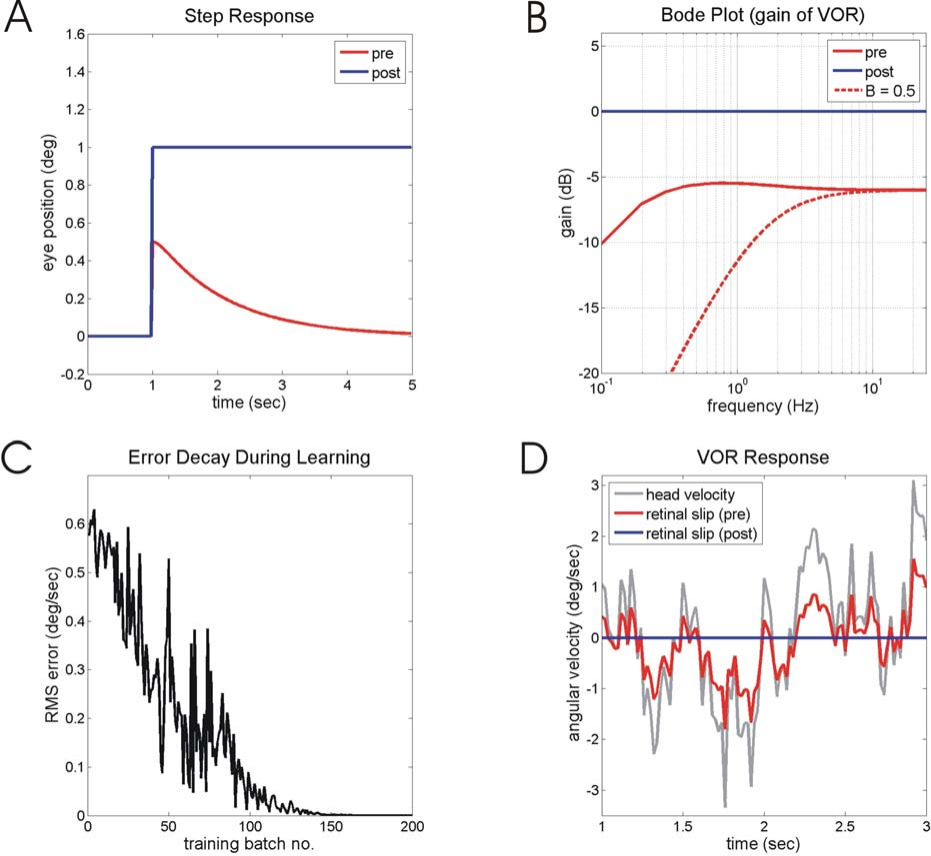
Performance of Model Before, During and After Training with an Undelayed Retinal-Slip Signal The plant *P* is first-order filter with time constant 0.1 s, and the brainstem controller B has an undergained (50%) direct pathway and a leaky integrator (TC = 1 s) in the indirect pathway (details in Methods). (A) Eye-position response to sudden head displacement. The desired and post-training performances are effectively identical, so that only the latter is shown. (B) System gain for sinusoidal input signals as a function of frequency (Bode gain plot). Gain is measured as ratio of eye velocity amplitude to head velocity amplitude. Performance before training is shown both for the complete brainstem controller (“pre”), and for the brainstem controller as simple gain (“B = 0.5”), which corresponds to the direct pathway on its own. After training, the desired and post-training performances overlap and only the latter is shown. (C) Decline in retinal-slip amplitude with training. Root-mean-square (RMS) retinal-slip amplitudes, measured over a 5 s training batch, plotted against number of training batches. (D) Example of retinal-slip to mixed-frequency head-velocity input before and after training.

Figure 3 illustrates compensation for a plant (*P* in Figure 2A) that can be approximated by a single linear viscosity and elasticity in parallel (first-order plant, time constant = 0.1 s). The dynamic characteristics described above are illustrated in Figure 3B, where the red dotted line shows the performance of the system when the brainstem controller *B* is a simple gain (and the cerebellum *C* is inoperative). The gain of the system as a whole is essentially constant above 10 Hz, but declines at lower frequencies, markedly so for frequencies below 2 Hz.

This brainstem controller corresponds to the “direct pathway” identified in the VOR by Skavenski and Robinson [24], which relays the head velocity signal to the motoneurons through a gain (in the example shown in Figure 3, this gain is set too low at 0.5 rather than at 1.0). Skavenski and Robinson also identified an “indirect pathway” which integrates the head-velocity signal to compensate for the plant's elasticity. In the simulations shown in Figure 3 the integrator in the indirect pathway is leaky with time constant ∼1 s, as suggested by the effects of floccular lesions (see Methods). The effects of the undergained direct pathway and leaky integrator can be seen in Figure 3A, 3B, and 3D (red line labeled “pre” in each). The eye movement in response to sudden head displacement (Figure 3A) is too small, and it returns to primary position within about 3 s. Continuous head movements of mixed frequency elicit eye movements that fail to eliminate retinal slip at either low or high frequencies (Figure 3D). The Bode gain plot (Figure 3B) shows a 5–6 dB loss of gain above ∼0.25 Hz, and a greater, frequency-dependent gain loss at frequencies below ∼0.25 Hz.

All these problems are remedied when the cerebellum is allowed to learn, using undelayed retinal slip as an error signal (Figure 3C). After learning, the compensatory eye movement to sudden head movement has the proper gain and is not followed by a drift back to the primary position (Figure 3A), and retinal slip following continuous mixed-frequency head movements is eliminated (Figure 3D). The plant compensation learnt in this manner is essentially perfect, as illustrated by the Bode gain plot of Figure 3B. The adaptive filter model of the cerebellum, embedded in a recurrent architecture, is thus able to learn both complex low-frequency plant compensation, and the high-frequency gain.

### Learning Impaired by Delayed Slip Signal

**Figure 4:**
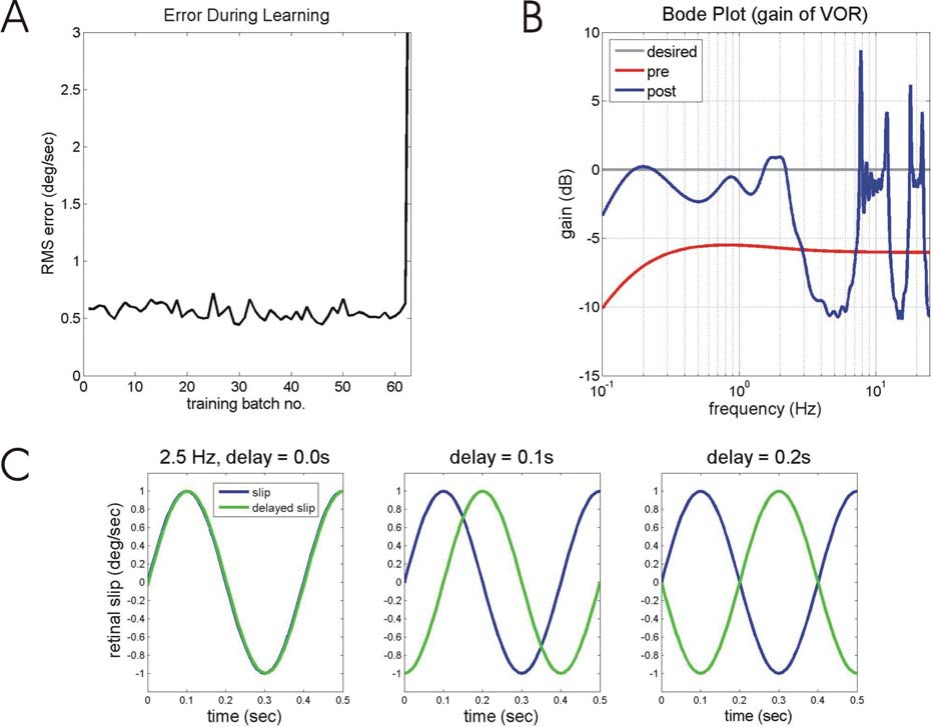
Model Performance Before, During, and After Training with a Retinal-Slip Signal Delayed by 0.1 s (A) Change in retinal-slip amplitude with training. It initially declines much more slowly than with an undelayed signal (Figure 3), and eventually increases very rapidly as the system becomes unstable. (B) System gain for sinusoidal input signals as a function of frequency, measured just before the instability shown in (A). The gains at frequencies above 2.5 Hz are inaccurate. (C) Effects of delay on correlation between two identical sinusoids at 2.5 Hz. As delay increases from a value of 0 s, the correlation declines from 1.0 to 0 at a delay of 0.1 s, and to −1.0 at a delay of 0.2 s.

The learning shown in Figure 3 is severely compromised if the simulated retinal slip signal is delayed by a realistic 100 ms (e.g., [25]). Instability can occur (Figure 4A), and the gains learnt at frequencies above 2.5 Hz (Figure 4B) are inaccurate. The reason for these problems can be seen in Figure 4C, which shows two sine waves of identical amplitude at 2.5 Hz, one delayed with respect to the other. At zero delay the correlation between the two signals is perfect (+1), but it becomes smaller as the delay increases, reaching 0 at 0.1 s delay when the two waves are 90° out of phase, and −1 at a delay of 0.2 s when the two waves are exactly out of phase. A learning rule that depends on the correlations between two signals will therefore become compromised if one of them is delayed. In particular, learning above the frequency at which the delay causes signals to become more than 90° out of phase may become unstable.

**Figure 5:**
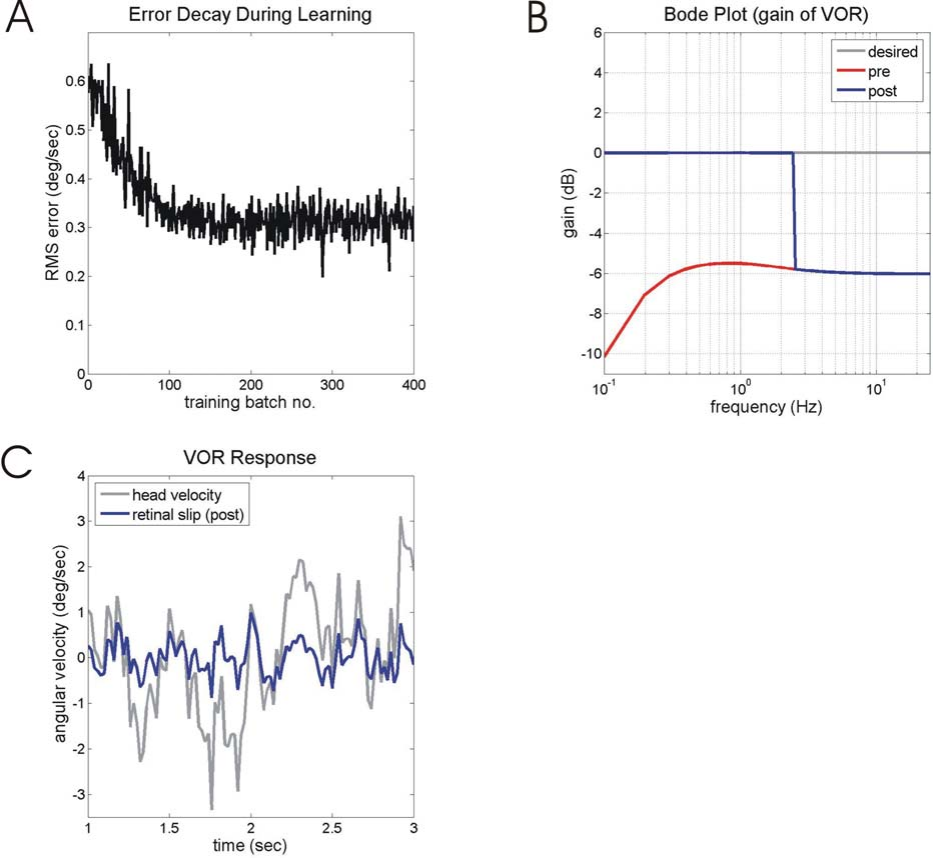
Model Performance with Delayed Retinal Slip and Frequencies >2.5 Hz Removed from Cerebellar Inputs (A) Learning is now stable, unlike that shown in Figure 4A, but with a greater asymptotic retinal-slip error than that shown in Figure 3C. (B) System gain for sinusoidal input signals as a function of frequency. No learning occurs for frequencies above 2.5 Hz. (C) Example of retinal-slip to mixed-frequency head-velocity input before and after training. Retinal slip is still present at frequencies above 2.5 Hz.

The above interpretation can be tested by removing any frequencies greater than 2.5 Hz from the modelled cerebellum. This was achieved in the model by having no such frequencies present in the parallel-fiber signal (Methods). Learning with such a constraint is stable (Figure 5A), but confined to frequencies below 2.5 Hz (Figure 5B) with a result that high frequencies remained in retinal slip (Figure 5C).

The behavior of the model shown in Figures 4 and 5 is much worse than that of the actual VOR, which remains accurate up to ∼25 Hz [21,22]. This discrepancy indicates a major computational problem for VOR calibration, namely how to maintain high-frequency performance when only a delayed error signal is available.

### Eligibility Trace Is Only a Partial Solution

Parallel fiber input to Purkinje cells is not only immediately summated to generate simple spike activity (Figure 1), but also generates postsynaptic changes in Ca^2+^ concentration (e.g., [28]). These changes may be long-lasting (∼400 ms) with a peak when parallel fiber activation precedes climbing fiber activation by ∼100 ms [39]. They are thought to assist contingency detection for events occurring ∼100 ms after the parallel fiber firing, and play a part in mechanisms of plasticity at parallel fiber synapses on Purkinje cells. The time course of the changes in Ca^2+^ concentration in effect delays and filters the parallel fiber input with respect to learning—the simple spike output which is needed for online control is not affected—and appears to correspond to the theoretical construct of a stimulus trace whose instantaneous amplitude indicates its current eligibility for inducing learning, (hence the term “eligibility trace”). An eligibility trace has recently been used in a model of the flocculus by Kettner et al. [40] to address the problem of the 100 ms delay in retinal slip signal for learning smooth pursuit trajectories, and has been discussed specifically in the context of VOR gain adaptation by Raymond and Lisberger [20]. We show here that the time courses regarded as plausible for the eligibility trace cannot extend gain storage in cerebellar cortex beyond about 10 Hz, so for higher frequencies above 10 Hz a brainstem site of plasticity is required even if an eligibility trace is present.

**Figure 6:**
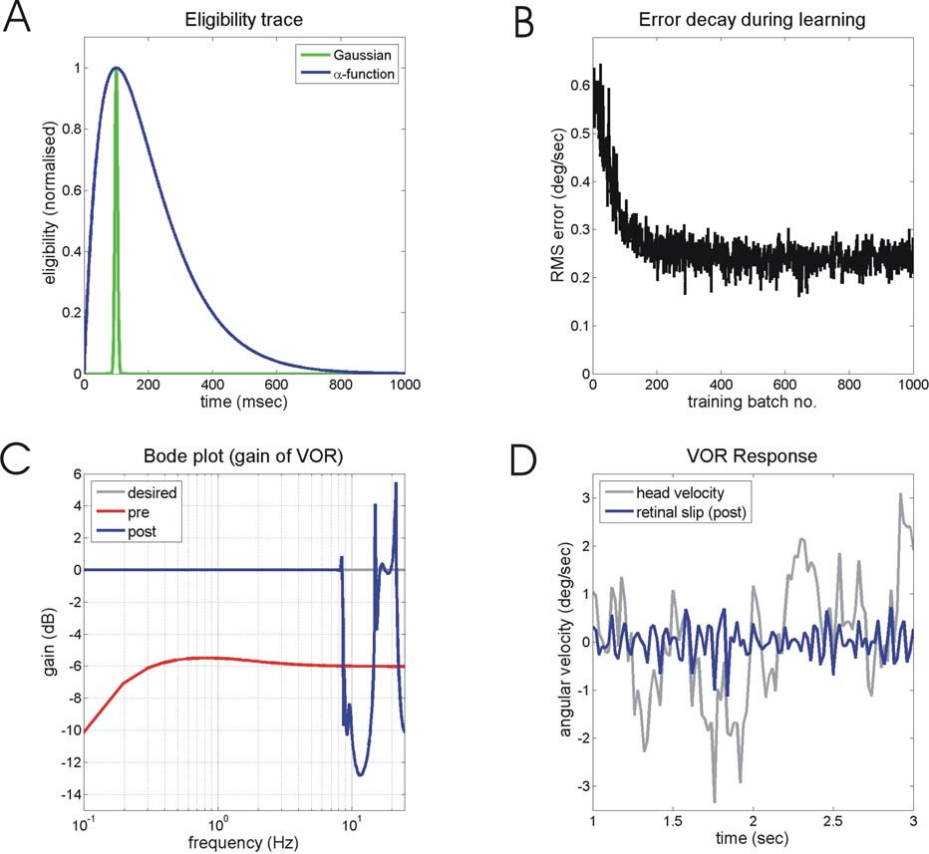
Effects of Eligibility Trace on VOR Calibration (A) Eligibility trace (blue line) used by Kettner et al. [40] and (B–D) compared to the trace required for VOR learning up to 25 Hz (green line). (B) Learning as measured by retinal slip error. (C) System gain for sinusoidal input signals as a function of frequency before and after learning. (D) Time course of retinal slip in response to colored-noise head-velocity input after learning.

Figure 6A (blue line) illustrates the time course of the eligibility trace used by Kettner et al. [40] for modelling how the flocculus could learn predictive smooth pursuit. The effects of incorporating this trace in the present VOR model are illustrated in Figure 6B–6D. Asymptotic retinal-slip error (Figure 6B) is slightly improved relative to Figure 5A, but accurate gains are only acquired for frequencies up to ∼8 Hz (Figure 6C), so that high frequency retinal slip is still present (Figure 6D).

Raymond and Lisberger [20] modelled the effects on learning of systematically varying the width of the eligibility trace (using a symmetrical Gaussian function rather than the asymmetrical alpha function illustrated in Figure 6A). To reproduce their experimental findings of good learning at a training frequency of 5 Hz and detectable learning at 10 Hz [25], a width at half amplitude of 60 ms was required. We found here that to extend good VOR learning up to 25 Hz requires a trace of the form shown as the green line in Figure 6A, with width at half the amplitude of 10 ms. Its narrowness would not be compatible with candidate molecular mechanisms of the eligibility trace, and in any case would not allow learning over the full range of delays at which climbing fiber signals arrive (see Discussion).

In summary, the addition of a plausible eligibility trace enables learning to occur at training frequencies higher than the 2.5 Hz limit otherwise imposed by the 100 ms delay in the retinal-slip signal. However, it does not allow learning for training frequencies above about 10 Hz.

### Brainstem Site of Plasticity Improves Performance

**Figure 7:**
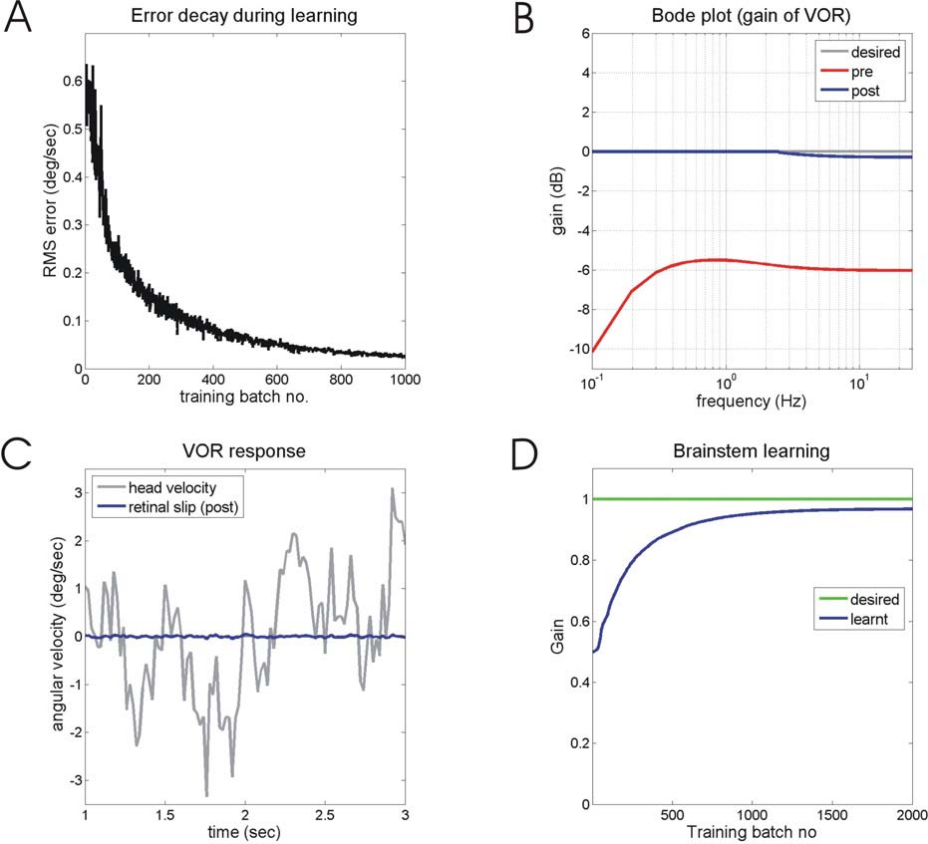
VOR Calibration with a Second Site of Plasticity in the Brainstem (A) Learning as measured by retinal slip error. (B) Bode gain of system before and after learning. (C) Retinal slip in response to colored-noise head velocity before and after learning. (D) Change in gain of brainstem as learning proceeds.

A second method of improving the poor high-frequency performance illustrated in Figure 5 is to include a brainstem site of plasticity. Learning in cerebellar cortex caused by changes in the plant produces cortical output that is correlated with vestibular input. In the simulation shown in Figure 7, learning in cortex was confined to frequencies below 2.5 Hz, as in Figure 5. Here, however, the correlation between vestibular and floccular input to the brainstem bandpassed to the frequency range 2.0–2.5 Hz (see Methods and the remarks below for the constraints on this frequency range) was used to alter the brainstem's intrinsic gain. Inclusion of this second site of plasticity improves learning (Figure 7A), final performance (Figure 7B), and residual retinal slip (Figure 7C). The gain of the overall system at 25 Hz is now 0.97, as a result of the gain change stored in the brainstem (Figure 7D).

Since the results illustrated in Figure 7 are central to the main argument of the study, it is important to establish how far they are robust rather than being the fortuitous outcome of a particular set of modelling parameters. Varying the parameters in the model showed that the system learned to produce accurate VOR gain at high frequencies (e.g., 25 Hz) (i) for either decreases or increases in plant gain, (ii) when a plausible eligibility trace was used, as illustrated by the blue line in Figure 6A from Kettner et al. [40], (iii) if the gain of the vestibular input to the brainstem was altered rather than the brainstem's intrinsic gain, (iv) if different basis functions were used for the decomposition of the mossy fiber input (Methods), (v) over a wide range of learning rates, provided the learning rate for the brainstem was substantially slower than that for cortex (to prevent oscillations in VOR gain during learning).

The parameters that were critical for good model performance were the combined dynamics of the brainstem *B* and plant *P*, in relation to the upper frequency limit at which the cortex could learn. For example, the combination of a first-order *P* (time constant 0.1 s) with *B* as simple gain produces the Bode plot shown in Figure 3B (labeled B = 0.5). The gain of the combination approaches its high-frequency value (−6 dB) at about 4–5 Hz. If the cortex can learn at this frequency, as it can with an eligibility trace, then a suitable approximation to the high-frequency gain can be stored in the brainstem. However, the actual task for the cortex is likely to be easier than this, because addition of a leaky integrator to *B*, as suggested by a wide variety of experimental evidence, produces a combination of *B* and *P* with a gain that approaches its high-frequency value at a much lower frequency (∼ 0.3 Hz, Figure 3B). In this case, the cortex could learn an appropriate value for storage in the brainstem at any frequency range above 0.3 Hz.

This analysis suggests that there is a substantial safety factor between the upper frequency at which the cortex can learn (5–10 Hz) and the upper frequency at which it needs to learn (0.3 Hz). Such a safety margin is important for the robustness of the model, especially as the dynamic characteristics of *B* nor *P* have yet to be precisely characterized. In fact the combination of a more realistic fourth-order plant model [41,42] with a leaky integrator brainstem controller (Methods) gives a system that also approaches its high-frequency value at ∼0.3 Hz.

### VOR Adaptation with 5 Hz Training

Comparison of the present model with experimental findings is complicated by the widespread use of methods for adapting VOR gain that mimic the effects of changes in vestibular input, rather than changes in the oculomotor plant (Introduction). Whereas plant changes affect all types of eye movement, methods that mimic vestibular changes do not affect the accuracy of smooth pursuit and related ocular tracking mechanisms such as the OKR. This accurate tracking can potentially affect VOR gain adaptation in at least two ways: (i) the retinal slip signal otherwise induced by inaccurate VOR gain will be reduced, and (ii) if the tracking is mediated by the flocculus itself, floccular output will be correlated with vestibular signals and could drive brainstem learning immediately, that is without any prior cortical learning (see Discussion).

**Figure 8:**
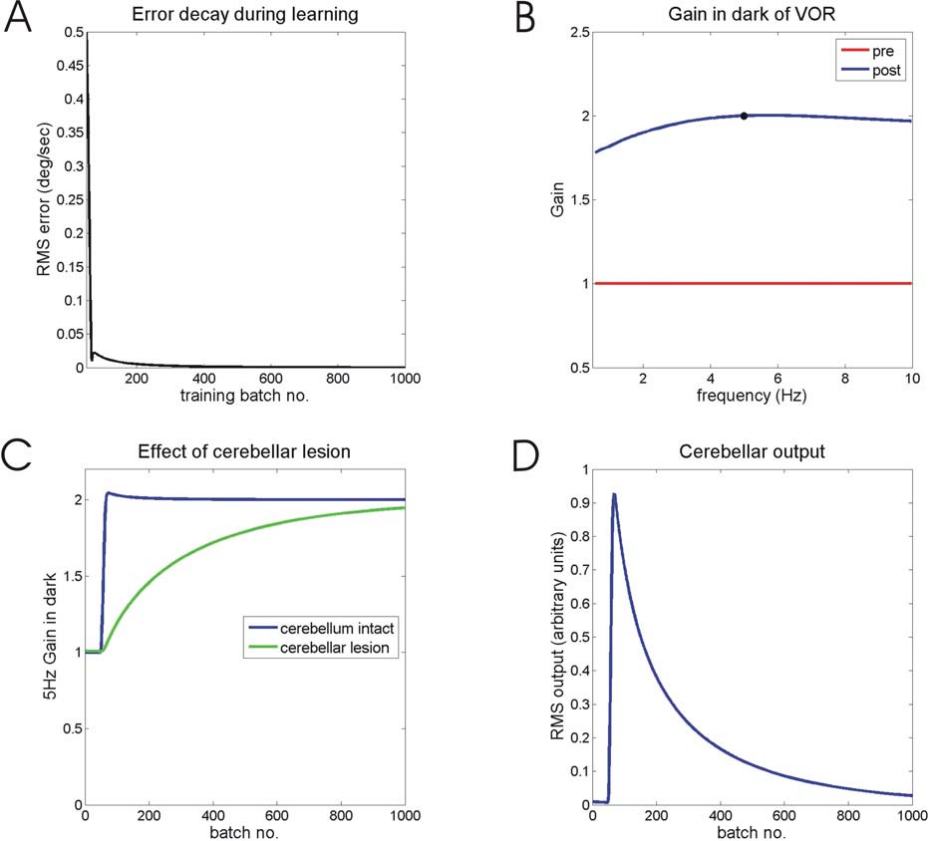
VOR Calibration with a Second Site of Plasticity in the Brainstem at 5Hz Training Frequency (A) Learning as measured by retinal-slip error. (B) Generalization of gain to frequencies other than that used in training. Graph format chosen to resemble that of Figure 7 of Raymond and Lisberger [25]. Black disc on “post” curve indicates training frequency. (C) Gain of system as training proceeds, with or without the simulated cerebellum. The effect of cerebellar inactivation becomes smaller with longer training. (D) Output of simulated cerebellum as training proceeds. An initial fast rise from a pre-training value of 0 is succeeded by a slower fall, eventually back to 0.

One way of avoiding these complications is to adapt the VOR in circumstances where tracking mechanisms are ineffective, for example by confining the training to a single high frequency such as 5 Hz [20,25]. Data from these studies were therefore compared with model performance when the simulated head-velocity frequencies were restricted to 5 Hz, as shown in Figure 8. Provided a suitable eligibility trace is used, in this case the one described by Kettner et al. [40] and shown in Figure 6A (blue line), the model is able to learn the required gain (Figure 8A). When adaptation is complete, the generalization of the new gain across frequencies (Figure 8B) is very similar to that found experimentally (Figure 7 in [25]) because the new gain value has been taught to the brainstem. Early in learning, however, the gain is stored mainly in cerebellar cortex, as illustrated by the effects of simulated floccular lesions (Figure 8C). These effects are consistent with the way in which simulated cerebellar output changes during training. Before training begins, cerebellar output modulation for 5 Hz head velocities is zero (Figure 8D), in accordance with experimental data on simple spike discharges for floccular Purkinje cells [20]. As training begins, cerebellar output modulation at 5 Hz increases sharply as a result of plasticity in cerebellar cortex. Later in training, cerebellar output modulation declines as a result of brainstem plasticity, until eventually it reaches zero once again as the correct gain value is learnt by the brainstem. This general pattern is observed providing that the brainstem learning rate is substantially slower than the cortical learning rate (10× slower in Figure 8). As mentioned above, high brainstem learning rates produce oscillations in system gain, the beginnings of which can be seen in Figures 8A and 8B. After about 20 training batches, the retinal slip error increases slightly (Figure 8A), corresponding to an overshoot in system gain (Figure 8B). It can be demonstrated analytically that the size of the overshoot is proportional to the ratio of brainstem to cortical learning rate.

One important feature of the learning illustrated in Figure 8 is that, because it takes place at a single high frequency, the cerebellar module *C* can learn using either the correlation between slip and efference copy (recurrent architecture), or between slip and vestibular input (forward architecture). The advantages of the recurrent architecture for learning plant compensation apply over the frequency range where gain and phase are frequency-dependent [11,12].

### Summary of Results

(i) The adaptive filter model of cerebellar cortex can learn to compensate for changes in both the dynamics and high-frequency gain of the plant provided the retinal-slip error signal is not delayed.

(ii) If, however, the error signal is delayed, learning by the adaptive filter becomes unstable above a frequency determined by the length of the delay. For a delay of 100 ms, this frequency is 2.5 Hz.

(iii) The instability can be prevented by removing frequencies above 2.5 Hz from the inputs to the filter, but this procedure prevents learning at frequencies greater than 2.5 Hz, whereas the VOR is accurate up to ∼25 Hz.

(iv) The upper limit for learning can be improved if the filter inputs used for learning are smoothed and delayed (the “eligibility trace”). However, the plausible upper limit for this process is ∼10 Hz, still substantially lower than 25 Hz.

(v) The correlation between filter (i.e., cortical) output and vestibular input over the frequency range 0.3–10 Hz can be used to drive plasticity at an additional (i.e., brainstem) site. With the appropriate learning rule, this additional site will learn a value close to the required gain of the reflex at frequencies above 10 Hz, so that VOR performance can reach the required level at 25 Hz.

These results are consistent with experimental observations that the VOR can only adapt to frequencies below ∼10 Hz, yet after adaptation they can perform up to ∼25 Hz.

## Discussion

It is generally accepted that VOR calibration requires at least two sites of plasticity, one in the flocculus and one in the vestibular nuclei [4,8,9,43–45]. Our previous analysis of VOR calibration suggested that the flocculus combined with an imperfect brainstem velocity-to-position integrator acts as an inverse model of the oculomotor plant, and that floccular plasticity is required to maintain the accuracy of that model in the face of plant changes [11,12]. This function can be achieved by an adaptive filter version of the basic Marr-Albus model of cerebellar cortex [10], embedded in a recurrent architecture that allows decorrelation of motor commands from retinal slip. The present study extends the computational analysis to address possible functions of the second, brainstem, site of plasticity.

The central problem is why a device as powerful as an adaptive filter should need an additional site of plasticity at all. Indeed, we demonstrate that in the linearised case such a filter is powerful enough to compensate for changes in both plant dynamics and high frequency gain, *provided the error signal* (*retinal slip*) *is not delayed.* However, a key element of the problem is revealed when the error signal is in fact delayed. We show that this introduces major stability problems in learning, which can be prevented by removal of high frequencies from the filter inputs only at the cost of degrading VOR performance at those frequencies. In short, the computational problem is identified as that of using a delayed error signal to adaptively calibrate high frequency performance. In the simplest case, the ∼100 ms delay of retinal slip precludes learning above 2.5 Hz, a serious deficiency given that VOR performance in primates remains good up to ∼25 Hz [21,22].

We then show that an algorithm for transferring a single gain value to a (brainstem) site outside the filter is able to solve this problem. The algorithm's success depends primarily upon the viscoelastic nature of the oculomotor plant, which means that above a certain frequency the plant can be treated as a simple viscosity with a fixed gain independent of frequency. Given plausible assumptions about the brainstem integrator, the algorithm is robust with respect to both modelling assumptions and plant parameters, and can in certain circumstances use alterations in cortical output driven by changes in parallel-fiber synapses conveying vestibular rather than eye-movement signals. The success of the algorithm illustrates why a brainstem site of plasticity may be required for VOR adaptation despite the power of the adaptive filter (cortical) model, and casts a new light on previous disputes concerning the implications of brainstem plasticity for Marr-Albus type models.

### Complexities of VOR Calibration

Although VOR adaptation was originally chosen as a relatively simple task for studying cerebellar function, subsequent experimental investigation showed it to be more complex than it first appeared. Commonly used adaptation paradigms (e.g., wearing goggles with magnifying lenses) do not affect the accuracy of the OKR or (in primates) smooth pursuit. At low frequencies (<∼2Hz) these tracking eye-movements can be used to follow the retinal slip generated during head movements, with two potentially important consequences. First, the retinal-slip error signal to cerebellar cortex may be reduced, perhaps very markedly. Second, because the flocculus itself generates pursuit commands, its output to the vestibular nuclei will be correlated with head-movement signals to those nuclei, and could therefore act as a gain error signal without any learning whatsoever occurring in the cerebellar cortex. In principle these two factors could combine to produce a “brainstem-only” learning mechanism for VOR adaptation [7], or a “brainstem-first” mechanism in which plasticity of parallel fiber synapses carrying vestibular signals is a secondary effect (in a direction opposite to that predicted by the original cerebellar learning models) with the function of ensuring stable eye movements in the face of the brainstem changes [43–46].

Because of these complicating factors, the involvement of a brainstem site of plasticity in VOR adaptation at low frequencies with these paradigms may not be at all informative about cerebellar cortical plasticity, especially if the putative error signal for that plasticity is reduced or eliminated by another oculomotor subsystem. To avoid the complications, the present study simulated circumstances where all eye movements were inaccurate, as would occur after change to the oculomotor plant. Since the VOR is a feedforward reflex, all of its components (Figure 1) require calibration. One of those components converts an eye-velocity command into the desired eye movement by taking into account the mechanical properties of the eye. Although this process of plant compensation is familiar in the oculomotor literature in the guise of the velocity-to-position integrator, it is sometimes regarded as quite separate from the VOR and the synaptic changes needed for VOR calibration. However the original concept of the neural integrator was formulated in the context of the VOR [24,27], lesion evidence indicates that part of the horizontal velocity-to-position integrator resides in the flocculus where the cerebellar cortical component of VOR plasticity is also thought to reside (e.g., [47,48]), and certain forms of VOR adaptation affect eccentric gaze holding [49,50]. As indicated above, a major advantage of modelling plant compensation rather than vestibular gain adaptation in the VOR is that the former affects all types of eye movements, whereas the latter leaves tracking movements intact which can potentially disable cortical learning.

We were able to show that a brainstem site of plasticity is still required even in the case of plant compensation. Learning occurs first in cortex (hence a “cortex-first” mechanism) and is transferred subsequently to the brainstem. The fact that a brainstem site of plasticity is needed even in simpler contexts than those usually studied powerfully demonstrates the difficulty of achieving high-frequency performance with a delayed error signal.

Given that the complications of VOR adaptation can be reduced by considering plant compensation, and that robust plant compensation requires the recurrent architecture (Figure 2A), this was the architecture chosen for the model. However, the study's conclusions regarding the need for a brainstem site of plasticity are independent of the choice of architecture. The unstable learning induced by delayed error signals applies just as much to a forward architecture which uses only vestibular inputs to the flocculus as does the remedy of brainstem plasticity. For example, if all eye movements are vestibularly driven, an adaptive filter can compensate for changes in vestibular gain with a simple feedforward architecture in which component *C* in Figure 2A receives from *V* only, with no feedback copy of the eye-movement command being required. Since this condition is approximated by training with 5 Hz stimuli (Figure 8), the conclusions concerning transfer of gain from cortex to brainstem are independent of whether cortical plasticity develops in vestibular or efference-copy pathways to cerebellar cortex.

### Evidence from Studies of VOR Adaptation

The above analysis suggests that brainstem plasticity driven by the correlation between floccular and vestibular inputs can arise in two different ways. It appears that these two mechanisms for inducing brainstem plasticity would have complementary functions, appropriate for different conditions. The brainstem-first mechanism would be important in circumstances when the retinal-slip error signal is markedly reduced by ocular tracking [18]. In contrast, the cortex-first mechanism proposed here would operate whenever there is sufficient retinal slip to drive cortical learning*.* This distinction points to the kind of experimental evidence relevant to deciding whether the mechanism proposed here actually exists.

#### VOR adaptation in the absence of accurate ocular tracking.

Accurate oculomotor tracking can be prevented by inducing or simulating changes in the oculomotor plant, so that plant compensation is tested directly. This method seems rarely to have been used. However, an alternative method for preventing tracking, in which training is confined to high frequencies where pursuit gain is very low, has been exploited in a series of studies by Raymond and Lisberger [20,25,51,52]. They were able to demonstrate clear adaptation of VOR gain after training with visual–vestibular mismatch at 5 Hz, even though pursuit gain is effectively zero at this frequency so that the subjects' eye movements were similar whether training was to increase or decrease VOR gain (p. 9217 in [20]). Moreover, whereas the responses of floccular climbing fibers did contain sufficient information to guide learning at 5 Hz, the simple spike responses of floccular Purkinje cells did not. These results suggest that at 5 Hz learning takes place first in cerebellar cortex rather than in the brainstem, consistent with the mechanism proposed here. In addition, gain changes induced at 5 Hz generalized well to the frequency range 0.2–10 Hz [25], implicating subsequent transfer of learning to the brainstem (Figure 8B).

Another possible source of evidence for the cortex-first mechanism might be whether VOR adaptation works in species where ocular tracking is less well-developed than in primate, for example in afoveate species. In fact, VOR adaptation appears to have been demonstrated in all species in which it has been examined, including cat, rabbit, mouse, and goldfish [4]. However, detailed measurement and modelling of both tracking capabilities and floccular output are needed to assess whether in any given experiment the actual tracking signal from the flocculus could or could not serve to induce brainstem plasticity. Similar considerations apply to the study of Ono et al. [53], which showed impaired smooth pursuit in primates (produced by unilateral lesions of dorsolateral pontine nuclei) was not associated with any discernible effect on VOR adaptation.

#### Order of learning in VOR adaptation.

As its name implies, in the cortex-first mechanism the floccular signal for inducing brainstem plasticity is itself produced by prior cortical plasticity. In contrast, the floccular signal in the brainstem-first mechanism is immediately available as soon as VOR adaptation starts. This distinction points to additional sources of evidence relevant to the existence of the cortex-first mechanism.

One is the effects of floccular inactivation. It is generally agreed that although very large cerebellar lesions do not systematically affect VOR gains above ∼1 Hz , adaptation of VOR gain can be completely prevented by much more restricted inactivation centered on the floccular region. This pattern of evidence strongly suggests that the flocculus provides a signal that allows gains to be stored in the brainstem, but does not on its own indicate whether that signal itself has to be learnt, and therefore does not distinguish between the brainstem- and cortex-first mechanisms. The additional evidence needed is whether floccular inactivation does affect VOR gain, provided the gain has been adapted recently (cf. Figure 8C). Reviewing such evidence, Boyden et al. [4] conclude that “studies that reported small effects of lesions on the expression of previously acquired changes in VOR gain generally used longer training paradigms than did those that reported large effects. These results are consistent with the storage of motor memories initially depending on the cerebellum and with the storage of longer-term memories depending more upon other structures” (p. 592 in [4]). More recent experiments have also produced this pattern of results [54,55]. However, the technical challenges facing such studies are formidable, and it is not yet clear that alternative explanations of their results have been decisively ruled out [4].

A second source of evidence concerns the effects on VOR gain adaptation of treatments known to impair learning in cerebellar cortex. Examples include lesions of the nucleus of the optic tract, a structure that relays retinal-slip signals to the inferior olive [56], floccular micro-injections of appropriate pharmacological agents [54], and genetic manipulations that target cerebellar long-term depression [57]. All these treatments have severe effects on VOR gain adaptation, at least in the short term, consistent with the cortex-first learning mechanism proposed here. However, there remain questions about the extent to which these treatments affect ocular tracking, and about what we would expect from the brainstem-first mechanism if cortical plasticity were blocked. Once again, explicit modelling is likely to be needed to address these questions and to allow proper evaluation of the above treatments.

#### Conclusions.

Although it is generally agreed that there are two sites of plasticity in VOR gain adaptation, the order in which learning occurs has proved difficult to establish. Cortex-first learning, as required by the present modelling results, is consistent with the results of studies showing that (i) VOR gain adaptation occurs in afoveate animals with weak ocular following; (ii) floccular inactivation affects recently adapted VOR gains; (iii) treatments which block cortical learning impair VOR gain. However, the strongest evidence in favor of cortex-first learning is at present provided by the studies of Raymond and Lisberger demonstrating clear VOR gain adaptation after training confined to 5 Hz, a frequency at which smooth pursuit is inoperative and no floccular training signal for the brainstem is initially available. The cortex-first mechanism suggested here can simulate this learning (Figure 8). In addition, it predicts that floccular output would change as a result of early training, but then revert to its original form as training continued (Figure 8D). It may prove possible to test this prediction by appropriate recording from Purkinje cells in the flocculus.

### Implementation of Algorithm

Although the main purpose of the modelling approach adopted here was to clarify computational issues (Introduction), these issues themselves turned out to have implications for how the two-site algorithm might be implemented, particularly with regard to the eligibility trace and the nature of brainstem plasticity.

#### Nature of eligibility trace.

Our analysis of the simplest model of cortical plasticity suggested that the process needed to be filtered at 2.5 Hz to cope with a 100 ms delay in the retinal-slip signal. However, there is clear evidence for substantial learning at a training frequency of 5 Hz, and weak learning even at a training frequency of 10 Hz [25]. As explained in Results, one way in which the algorithm could learn at these frequencies is for parallel fiber signals to be delayed and filtered as part of the signaling mechanisms underlying synaptic plasticity (Figures 6 and 8). The result of delaying and filtering is usually referred to as an “eligibility trace.”

The idea of an eligibility trace seems to have originated in learning theory as the “stimulus/stimulating trace,” invoked to account for features of classical conditioning such as the variation in acquisition rate with the interval between the onset of conditioned and unconditioned stimuli [58]. It has recently been used in a model of the flocculus by Kettner et al. [40] to address the problem of the 100 ms delay in retinal-slip signal for learning smooth pursuit trajectories, and has been discussed specifically in the context of VOR gain adaptation by Raymond and Lisberger [20]. We show here that what are regarded as plausible shapes for the eligibility trace cannot extend gain storage in cerebellar cortex beyond about 10 Hz, so a brainstem site of plasticity is required even if an eligibility trace is present.

There are, however, a number of central issues concerning the eligibility trace that remain to be resolved, including its molecular basis (e.g., [3,38,39]), and how its properties could be consistent with findings on spike timing and plasticity in cerebellar slices. One particular problem is that a 100 ms delay in climbing fiber input is not characteristic of all regions of cerebellar cortex: for example, the delay may be as short as 10–15 ms for C1 zones [59]. It may prove possible to exploit the requirements for learning at different delays to constrain the shape of the functional eligibility trace [60].

#### Mechanisms of brainstem plasticity.

Both the brainstem- and cortex-first learning mechanisms discussed above use the correlation between cerebellar and vestibular inputs to the brainstem to drive its plasticity. They do not require brainstem collaterals of climbing fiber input to the flocculus, which in any case appear to be weak or nonexistent: the relevant regions of the inferior olive “do not project to the vestibular nuclei that are the main targets of floccular Purkinje cells” (p. 13 in [61]). There is evidence for plasticity in neurons in the vestibular nuclei, with respect to both intrinsic gain (e.g., [62,63]) and the efficacy of vestibular synapses on those VN neurons that receive input from the flocculus (e.g., [43,45]). At present the mechanisms whereby these kinds of plasticity could be driven by correlations in their inputs are under active investigation (e.g., [64,65]). Initial findings indicate that, as predicted by the present simulations, long-term depression can be induced in vestibular synapses on VN neurons in vitro, by combining stimulation of vestibular afferents with hyperpolarisation of the target neuron to mimic the effects of increased cerebellar input [66,67].

The present results raise the question of the frequency range over which the putative plasticity mechanisms would need to operate. In general terms, the bandpassed covariance rules used here can be satisfactorily approximated by spike timing–dependent plasticity rules combining a narrow central region of LTD with a wide lobe of LTP. However, modelling individual vestibular neurons at a more detailed level is needed to provide more precise predictions concerning both spike timing–dependent plasticity [68] and frequency ranges. Further data are also needed to address the question of whether there are additional correlated inputs to the vestibular nuclei that could drive plasticity in the appropriate circumstances.

### Relation to Previous Models of VOR Adaptation

There are two features of the present modelling approach that together determine its relation to previous models of VOR adaptation. One is that it addresses the functional requirements of VOR calibration by simplifying the problem as much as possible, while retaining its key signal-processing features. The second is that it focuses on VOR calibration in response to changes in the oculomotor plant, rather than to changes in vestibular processing (see section on Complexities of VOR Adaptation). By combining these two features, the present model is, to our knowledge, the only one that can use a delayed error signal to learn gains at low training frequencies and then to perform accurately up to 25 Hz.

In relation to previous models, the present approach is first of all complementary to detailed descriptive models of VOR adaptation that seek to specify what kinds of synaptic or other local changes are needed to account for the electrophysiological and behavioral changes observed after VOR gain adaptation (e.g., [44,69,70]). These models have been important in demonstrating how the experimental data can only be explained by the assumption of plastic sites in both cerebellar cortex and brainstem. By incorporating learning rules and a functional model of cerebellar cortex, the present study complements this approach both by showing why a second site of plasticity in the brainstem is needed, and by describing a (cortex-first) mechanism for coordinating the two sites.

Second, as indicated above, the present approach is complementary to models that incorporate smooth pursuit as well as VOR adaptation, which enables them to examine the consequences of brainstem-first learning for eye movements and subsequent cortical plasticity [46].

Third, it is more general than systems-level models of VOR adaptation that ignore plant dynamics and hence the frequency-dependent nature of VOR gain [71–73]. It is not clear whether the algorithms proposed in these models would be able to generate the partial forward model of the plant required for VOR calibration.

Finally, the model used here resembles a model of the cerebellum developed for a different behavioral context, namely eyeblink conditioning [74,75]. Both models use a form of adaptive filter to represent cerebellar cortex, both use two sites of plasticity (one in the deep cerebellar nuclei, in the case of eyeblink conditioning), and in both learning occurs first in cerebellar cortex. The eyeblink conditioning model differs from that used here by representing individual neurons, which has the advantage of allowing more detailed predictions concerning single-unit electrophysiological data, but the disadvantage of making it harder to discern the overall mathematical functionality of the model. The issue of whether eyeblink conditioning in fact *requires* an extracortical site of plasticity is addressed briefly in the next section.

### Generalization to Other Adaptive Control Problems

Because the same basic microcircuit is involved in all cerebellar functions, the findings here from VOR calibration have potential relevance for all tasks in which the cerebellum is involved. This point is indeed one of the main justifications for studying cerebellar function in the VOR in the first place. Here we argue that a brainstem site of plasticity is required in VOR calibration because of the 100 ms delay in the retinal-slip signal that drives learning via climbing fiber input to the flocculus, and that the brainstem site need only store a simple gain because the oculomotor plant is viscoelastic in nature. In considering whether an extracortical site of plasticity might be a widespread feature of cerebellar operation, two issues have therefore to be considered. The first is that limbs have inertia as well as viscoelasticity, so that their Bode gain may not flatten out in a simple fashion at high frequencies. The second is that some cerebellar regions appear to receive climbing fiber inputs at much shorter latencies than those observed for the flocculus, e.g., 10–25 ms for tactile climbing fiber inputs to cat forelimb regions of anterior lobe (pp. 258–260 in [2]). These points raise the possibility that two sites of plasticity are only required in special cases, and are not a generic feature of cerebellar organization.

However, further considerations suggest that such a conclusion would be premature. In the VOR, the important dynamic properties were those of the plant and brainstem controller combined. In the case of limb control, this combination must be extended to include spinal mechanisms. It is possible that proprioceptive feedback presents the cerebellum with a plant that appears to be viscoelastic, in similar fashion to its suggested role in making the plant appear linear [76]. If so, the storage of high-frequency gains in the deep cerebellar nuclei might prove to be a feasible control strategy.

In the case of short-latency tactile climbing fiber signals, the example of eyeblink conditioning is particularly informative. The relevant areas of cerebellar cortex receive climbing fiber signals related to periorbital stimulation with latencies of 9–12 ms in cat [77]. But the amplitude of the unconditioned blinks evoked by such stimulation is itself under adaptive control, apparently by the same area of the cerebellum necessary for eyeblink conditioning [78]. This area may therefore receive an additional climbing fiber signal that is related to blink completion rather than blink initiation. Since little is known about this putative signal, including its latency, caution must be exercised concerning the computational requirements for an extracortical site of plasticity in eyeblink conditioning. This point illustrates a general conclusion that can be drawn from the present study, which is the importance of understanding the computational nature of the particular control task that is being used to investigate the cerebellar algorithm.

### Predictions

The “cortex-first” mechanism proposed here makes a number of predictions about VOR calibration. (i) When ocular tracking is prevented, for example by adapting VOR gain at 5 Hz, floccular Purkinje cell firing should at first become increasingly modulated in relation to the VOR. As training proceeds, however, this modulation should then start to decrease, and eventually disappear altogether as the new value of gain is stored in the brainstem. (ii) Treatments intended to target cortical learning, for example genetic manipulations that block cortical long-term depression, will be more effective the more the VOR calibration task is designed to preclude ocular tracking and hence brainstem-first learning. A recent study of VOR adaptation in mice with impaired cerebellar long-term depression (caused by absence of Ca^2+^/calmodulin-dependent protein kinase IV) appears to be consistent with this prediction, in that adaptation impairments were observed at high but not low training frequencies [79]. (iii) Plasticity in the vestibular nuclei induced by VOR calibration will be driven by the *correlation* between floccular and vestibular inputs, particularly for inputs at frequencies between 0.3–5 Hz.

In addition, the cortex-first mechanism makes a prediction about cerebellar learning tasks other than VOR calibration. For these other tasks, the importance of plasticity outside the cortex—primarily in the deep cerebellar nuclei—will depend on the exact nature of the task. To the extent that it involves substantially delayed error signals and good performance at high frequencies, such sites are likely to be very important.

### Issues for Future Modelling

As outlined above, a major task for future modelling of VOR calibration using the present computational approach is the incorporation of tracking eye movements, and their effects in both reducing retinal-slip error and inducing correlations between cerebellar and vestibular inputs to the brainstem. Such an extension of the present model would allow it to address the relative contributions of cortex-first and brainstem-first learning to VOR adaptation in circumstances where accurate tracking movements make a significant contribution. Understanding the relative contributions of these components is essential for interpreting the complex effects of frequency-selective adaptation in primates at frequencies below 5 Hz, both behavioural [25,80] and with respect to cortical plasticity [46]. It might then be possible to use the bandwidth of frequency-selective adaptation, as a function of frequency, to throw light on the nature of the basis filters used in the model.

A further important extension of the model is the incorporation of known nonlinearities. For example, there is evidence suggesting that the retinal-slip signal gives information only about slip direction, not velocity [18]. We have previously shown that a simple binary retinal-slip error signal is adequate for plant compensation in the otherwise linear model [11], but have not investigated the case of a binary signal that drops again to zero at high velocities. Such an error signal might have the advantage of preventing fast movements such as saccades or quick-phases from producing inappropriate learning during VOR adaptation. However, it is important to check that there are no plant compensation errors that only appear at high slip velocities, and so would never be registered by the error signal.

### Conclusions

As outlined in the Introduction, the modelling approach used here seeks to simulate only the essential signal-processing features of both the control task and the neural machinery that implement the algorithm for carrying it out [11,12]. In the present case, the approach has value in highlighting the combination of delayed error signal and good high-frequency performance that requires a brainstem site of plasticity, together with the particular dynamic properties of the plant and brainstem that enables such a site to be effective. The fact that an extracerebellar site of plasticity is required in such a stripped-down system suggests that it should be a generic feature of plant-related VOR calibration across species, independent of oculomotor features such as the presence of a fovea, frontally directed eyes, or smooth pursuit. Current experimental evidence offers some support for this view, suggesting that VOR calibration involves a brainstem site of plasticity in a wide range of vertebrates, including mice and goldfish.

The issue of sites of plasticity in the VOR has a long, complicated, and disputatious history, summarized in a recent review [4] as follows: “During the last several decades, attempts to discriminate between the Marr-Albus-Ito and Miles-Lisberger models have dominated research on motor learning in the VOR. Each model can explain some of the data regarding motor learning in the VOR with a single plasticity mechanism. However, several lines of evidence indicate that multiple plasticity mechanisms contribute to the regulation of this simple behaviour” (p. 602 in [4]). The contribution of the present study to this history is to indicate why the existence of a brainstem site of plasticity in VOR calibration does not falsify Marr-Albus type theories, but is in fact required by them.

## Methods

The model architecture shown in Figure 2A was programmed in MATLAB. *V*, *P*, *B*, and *C* were treated as linear processes, allowing use of functions in the Control Systems toolbox. The characteristics of the linear processes used in initial training were as follows.

(i) *V* was a unit gain.

(ii) *P* was a first-order plant, with the transfer function *H_p_*(*s*) between eye-in-head velocity *e_h_* and motor command *y* given by

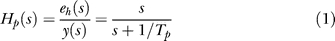
where *s* denotes the Laplace complex frequency variable and *T_p_* the time constant of the plant. The value of *T_p_* was set to 0.1 s [81]. (In subsequent equations with transfer functions, their argument (*s*) is omitted for simplicity).

(iii) The brainstem *B* had the transfer function *H_b_* given by

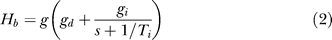
(where we set *g* = 1, see below) corresponding to a brainstem controller with two components [24]: (a) a direct path which passes the head-velocity signal to the plant with gain *g_d_*; and (b) an indirect path which acts as a leaky integrator with gain *g_i_* and time constant *T_i_*. This *H*_b_ is an exact inverse for *H*_p_ when *g_d_* = 1, *g_i_* = 1/*T_p_*, and the integrator is exact: *T_i_* = ∞. In simulations, both the gains were altered (usually to half their exact value) and the leaky integrator time constant was taken as *T_i_* = 1 s, as indicated by the effects of floccular lesions in primates [47]. In the simulations to be described here, this brainstem controller is separated into a fixed component *g_d_ + g_i_ /* (*s + 1 / T_i_*) and an intrinsic gain *g* (the multiplicative factor in Equation 2 above) which is assumed to be adaptive.

(iv) In adaptive filter models, *C* analyses the input *y*(*t*) into many parallel fiber signals *p_i_* = G_i_*y(*t*) which are resynthesised to form the output *z* = Σ *w_i_ p_i_*. In engineering applications, the basis filters *G_i_* are often taken to be tapped delay lines. It is clear that any complete basis of filters will give the same endpoint for learning; however, learning can be made significantly faster by choosing a basis such that the signals *p_i_*(*t*) are statistically independent [e.g., [11,82]). In the simulations, we use a sinusoidal basis [83] for which the signals *p_i_*(*t*) are the Fourier components of the input signal *y*(*t*) over the 10 s batch at a time resolution *dt* = 0.02 s. This basis maximises the rate of learning and corresponds to finely tuned channels whose width is the Nyquist limit. It allows us to recover theoretical limits on performance accurately and efficiently. In some simulations, we also used a more biologically plausible basis corresponding to a small number of overlapping coarsely tuned channels obtained by forming optimal linear combinations of six leaky integrators with time constants between 0.01 and 1 s.

The learning rule at the cortical site of plasticity was:

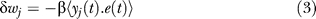
where *δw_j_* was the change in the *j*th weight *w_j_*, *β* a learning rate constant, *e*(*t*) the value of retinal slip at time *t*, *y_j_*(*t*) the value of the *j*th filter signal at time *t*, and <> denotes the expected value of the enclosed quantity over the time period used for training. The value of *β* was adjusted to give rapid learning without instability. Specifying learning rate bounds is not simple, since they depend on the nature of the inputs, the basis *G_i_*, etc. Although analytical rules can be found in the literature, they are rarely employed in practice since it is usually straightforward to find reasonable values.

The learning rule at the brainstem site of plasticity was:

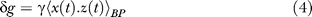
where *δg* was the change in the intrinsic gain *g* of the brainstem (see Equation 2). The positive learning rate *γ* was chosen such that learning at this site was significantly slower than at the cortical site of plasticity. The positive sign is correct for the sign convention shown in Figure 2. The effect is that intrinsic gain is reduced when excitatory vestibular inputs to brainstem are positively correlated with *inhibitory* cerebellar inputs. Here <>_BP_ denotes the expected value of the enclosed product with the signals bandpassed to an appropriate frequency range. The bandpassed learning rule ensures that the gain transferred from cerebellum to brainstem is the average gain over the pass band (*f*_min_ *, f*_max_). The lower limit *f*_min_ is fixed by the frequency down to which the plant gain (partially compensated by the brainstem) can be taken to be approximately constant (see Results for more details) and the upper frequency *f*_max_ is fixed by the high frequency limit of cerebellar learning since the cerebellum should not transfer gain at frequencies where it cannot learn accurately.

The training input to the system was a head-velocity signal modelled as colored noise with unit power. The power had its peak value at 0.2 Hz, then varied with increasing frequency *f* as 1/*f*. This head-velocity signal is intermediate in high frequency content between white noise acceleration and white noise velocity and has the advantage of providing plots in which the presence of a range of frequencies is obvious. As long as all frequencies are present in the head-velocity stimulus, its precise form only affects the speed of learning and not the result. For example, the colored noise can be replaced by sequential step responses with the same results. For efficiency, the weight-update rules in Equation 3 and 4 were implemented in batch mode using 10 s batches of head-velocity data. In Equation 4 the data were bandpassed over the appropriate frequency range before expectations were computed. Performance of the system was assessed (i) from the *e*(*t*) produced by the model, (ii) by applying a step head-position profile to the trained model, and (iii) by examining the Bode gain plot of the learned VOR transfer function between 0.1 and 25 Hz.

## Abbreviations

OKR: optokinetic reflex
VOR: vestibulo–ocular reflex
